# Locking plate fixation with fibular allograft augmentation for failed peritrochanteric fractures: A technical note

**DOI:** 10.1097/MD.0000000000046572

**Published:** 2026-01-02

**Authors:** Min Uk Do, Kyeong Baek Kim, Hyun Tae Koo, Won Chul Shin

**Affiliations:** a Investigation Performed at the Department of Orthopedic Surgery, Research Institute for Convergence of Biomedical Science and Technology, Pusan National University Yangsan Hospital, Pusan National University School of Medicine, Yangsan, Republic of Korea.

**Keywords:** fibular allograft, intertrochanteric fracture, nonunion, subtrochanteric fracture

## Abstract

The failure cases of peritrochanteric fractures are occasionally encountered due to poor bone stock. For proximal humeral fractures, a strategy using a segment of the fibular allograft (FA) placed endosteally was introduced. Several studies have demonstrated favorable outcomes with FA. Inspired by this, a fixation strategy using FA with a locking plate was performed in patients with unfavorable bone stock conditions. Ten patients with poor bone stock and nonunion of peritrochanteric fractures were treated with FA and a locking plate at a tertiary hospital between 2016 and 2022. We analyzed the bone union status, the time taken for union, and postoperative complications. Bone union was achieved in all cases following average time of 9.6 months (range 6–14 months). There was no complication related to allograft such as infection. The use of FA in conjunction with a locking plate for fixation in nonunion of peritrochanteric fractures with poor bone stock or cortical bone defects demonstrated successful outcomes in our study. Ongoing observations and reporting are essential for comprehensive validation and addressing potential complications.

## 1. Introduction

The incidence of hip fractures has markedly increased in recent years with an aging of population.^[1]^ A considerable proportion of hip fractures are intertrochanteric or subtrochanteric. Internal fixation is the treatment of choice for the types of fractures mentioned above.^[2–6]^ Although implants have been developed, failure cases are occasionally encountered owing to poor bone stock, unfavorable fracture patterns, inappropriate choice of implants, or improper implant position.^[7–10]^

Two situations must be overcome in cases of failed peritrochanteric fracture. One is implant failure, and the other is nonunion. Problems associated with implant failure necessitate decision-making regarding the removal of the internal fixation device and the method for refixation. In the nonunion site, the associated challenges include overcoming deformities at the fracture site and restoring alignment. Additionally, addressing bone grafting at the nonunion site and overcoming bone defects become crucial issues.

A high incidence of screw perforation, varus collapse, and fixation failure has been reported in medial column comminuted proximal humeral fractures.^[11,12]^ A strategy for providing medial support using an endosteally placed segment of the fibular allograft (FA) was introduced. Several studies demonstrated favorable outcomes with FA.^[13–15]^ Inspired by this, a fixation strategy using FA with a locking plate was performed in patients with unfavorable conditions for bone stock. We introduce this method because it yields positive outcomes.

## 2. Methods

The patient information was reviewed by the University Human Subjects Committee, and an informed consent exemption was obtained from the institutional review board (IRB) of our affiliated institutions (Pusan National University Yangsan Hospital, Approval No. 55-2024-046). A locking plate fixation with FA was performed in 10 cases at a tertiary hospital from 2016 to 2022. These patients had undergone multiple operations in another hospital or had large bone defects compromising internal fixation, along with poor bone stock. This technique was performed for nonunion of peritrochanteric fractures. Six patients had nonunion of the subtrochanteric fracture, and four had nonunion of the intertrochanteric fracture. An experienced orthopedic surgeon performed these procedures using Non-Contact Bridging Periprosthetic Femur Plate System (Zimmer Biomet, Warsaw), Non-Contact Bridging Distal Femur Plating System (Zimmer Biomet), or LCP Distal Femur Plate (DePuy Synthes, Zuchwil, Switzerland).

### 2.1. Case illustrations

#### 2.1.1. Case: a nonunion of intertrochanteric fracture

**Figure 1.**
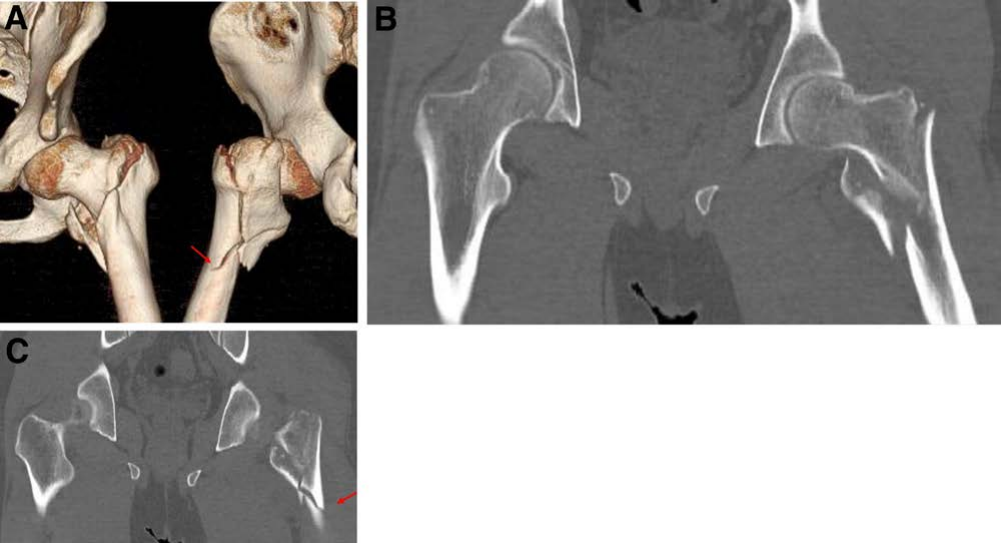
Computed tomography (CT) images showing (A) a 3D reconstruction and (B and C) coronal views of an intertrochanteric femoral fracture (AO/OTA 31-A3) in a 38-year-old patient (red arrow).

**Figure 2.**
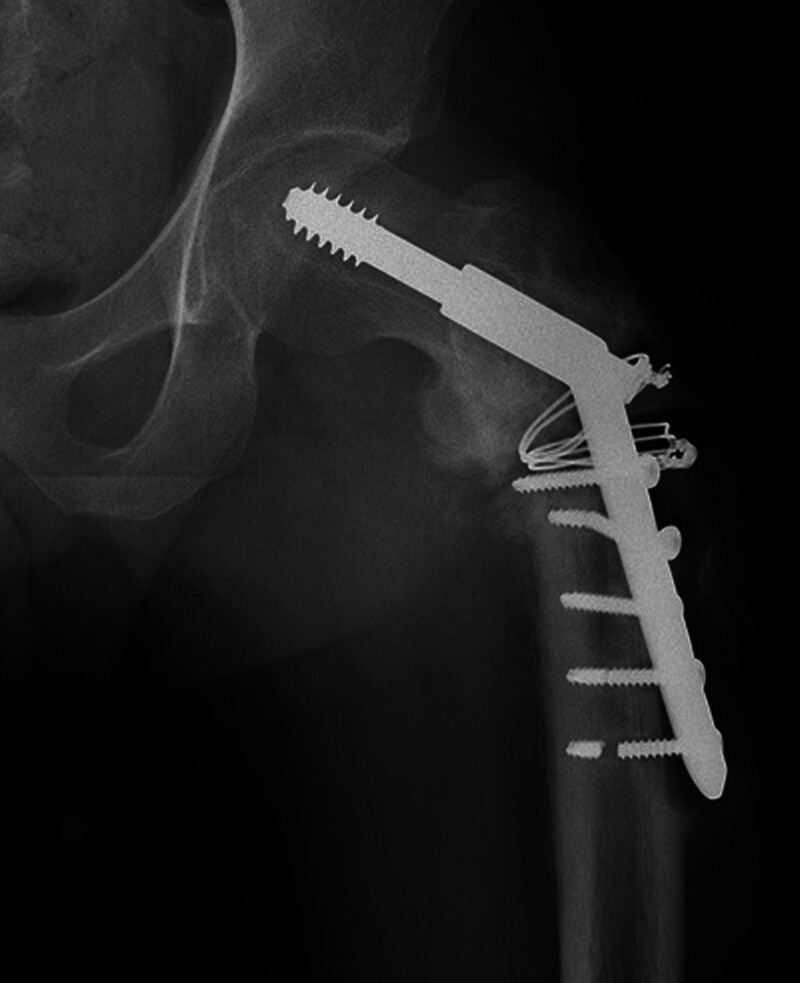
Radiograph displaying nonunion and implant failure of intertrochanteric fracture with broken screws.

A 38-year-old male patient with an AO Foundation/Orthopedic Trauma Association A3-type unstable intertrochanteric fracture presented with a transverse fracture line involving lateral wall as observed in the computed tomography scan (Fig. 1). The patient had previously undergone open reduction and fixation with compression hip screw and wiring at another hospital. After 8 months, he visited our clinic, revealing nonunion and implant failure with a broken screw (Fig. 2). Radiographic examination showed a radiolucent line around the implant, severe bone erosion, and bone defect between the plate and the lateral cortex of the femur. To rule out infection, laboratory evaluation, including C-reactive protein and bone scan, were conducted. Bone graft and fixation using a locking plate were considered preoperatively.

### 2.2. Surgical technique

**Figure 3.**
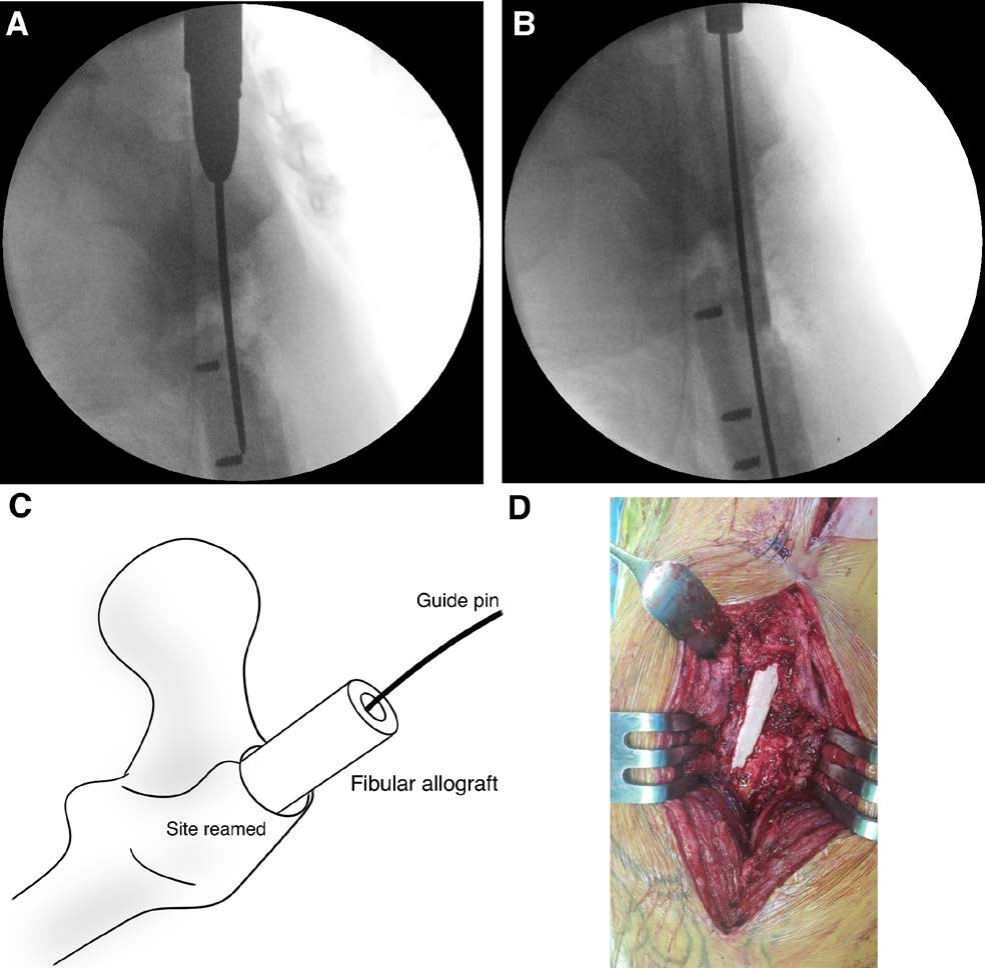
(A) Entry is established at the greater trochanter tip using the proximal femoral nail instrument. (B and C) The fibular allograft (FA) is transferred into the femur canal along with the guide wire. (D) An intraoperative photograph showing FA within the intramedullary space serving as a cortical support.

A direct lateral incision was made through the previous operative scar. Specimens from the implant area were collected for frozen biopsy and bacteriologic testing to confirm the absence of infection. Neutrophils were not observed in any of the frozen biopsy specimens. The implant was removed (non-interfering parts were left in place). The lateral cortex defect, which was severely compromised where it attaches to the plate, had no corresponding near cortex for plate fixation. Thorough removal of fibrous tissue at the nonunion site was performed, confirming a healthy bone margin with visible bleeding. Using the proximal femoral nail instrument, entry at the greater trochanter tip was established using C-arm images (Fig. 3A).

On the fracture table, the nonunion site was approached and manipulated for alignment and length correction. A sharp tip long-guide was inserted through established entry on the greater trochanter (sharp tip long-guide instead of ball tip guide for removal after the FA insertion). If the reamer is not fixed with a drill, a ball tip guide was initially used and later replaced with a sharp tip. Intramedullary reaming for internal bone graft and fibular insertion was meticulously performed, especially at the nonunion site. Reaming size was confirmed, and an appropriately FA was prepared (trimmed to match reaming size or slightly larger, by approximately 0.5 mm, ensuring sufficient length above and below the nonunion site for effective intramedullary support, such as an intramedullary nail).

**Figure 4.**
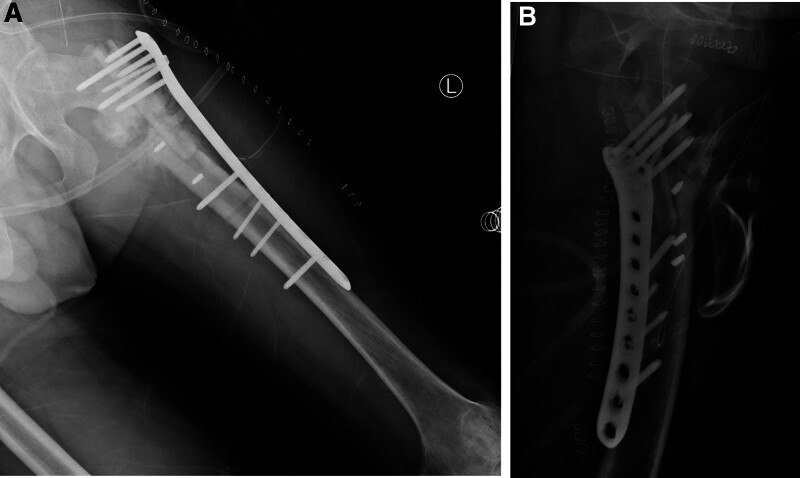
(A) Plain anteroposterior (AP) radiograph (B) lateral radiograph taken postoperatively after plate fixation with FA.

The guidewire was positioned within the fibular canal and transferred into the femur canal using an appropriate impactor guided by C-arm images (Fig. 3B and C). In cases with lateral wall defects, the FA within the intramedullary space can serve as cortical support for plate fixation (Fig. 3D). Subsequently, a proximal femur anatomical plate, in this case, utilizing the opposite LCP Distal Femur Plate (DePuy Synthes), was positioned and fixed. During fixation, precautions were taken to avoid fracturing the fibula, and if possible, locking screw fixation was applied, with as many screws fixed proximally as possible. For compression at the nonunion site, compression screws were inserted initially, followed by a final replacement with locking screws. Autogenous cancellous bone harvested from the ipsilateral iliac crest was grafted at the nonunion and defect sites (Fig. 4).

### 2.3. Postoperative care

On the second postoperative day, the patient was instructed to walk with partial weight-bearing using crutches. There was no difference in postoperative rehabilitation methods and medication with other proximal femur fracture patients. The postoperative radiologic review was performed at 6 weeks; 3, 6, and 12 months; and annually thereafter.

## 3. Results

**Figure 5.**
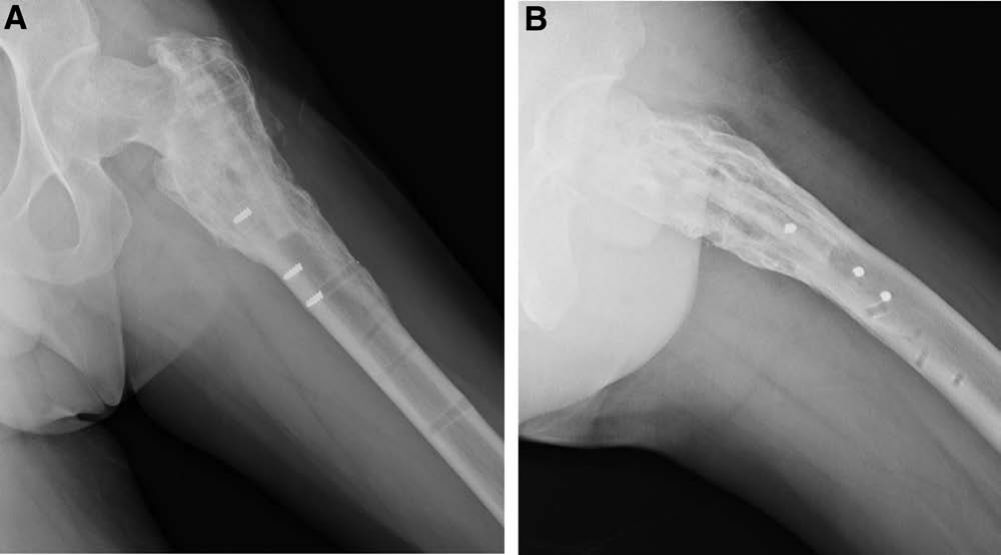
(A) Plain anteroposterior (AP) radiograph (B) lateral radiograph obtained after implant removal (3 years after fixation) display a union of intertrochanteric fracture.

Ten patients with nonunion of peritrochanteric fractures, who has poor bone stock or cortical bone defect, were operated with FA. Bone union was achieved in all cases (100%) after an average time of 9.6 months (range 6–14 months) (Fig. 5). Mean follow-up period was 19 months (range 8–36 months). Non-Contact Bridging Periprosthetic Femur Plate System (Zimmer Biomet) was used in four cases, Non-Contact Bridging Distal Femur Plating System (Zimmer Biomet) was used in a case, and LCP Distal Femur Plate (DePuy Synthes) was used in 5 cases. There was no complication related to allograft such as infection.

## 4. Discussion

Peritrochanteric fractures are occasionally encountered owing to poor bone quality.^[7–10]^ Nonunion rates were 2% for intertrochanteric fractures and 7% to 20% for subtrochanteric fractures.^[16–19]^ Studies suggest successful outcomes in intertrochanteric nonunion with intramedullary or extramedullary fixation.^[8,20]^ For subtrochanteric nonunion, intramedullary fixation yielded a 98% union rate, and extramedullary fixation achieved a 95% union rate.^[21]^ Despite successful outcomes of peritrochanteric nonunion, high-level evidence for optimal surgical management is lacking. Additionally, no study specifically addressed peritrochanteric nonunion in patients with poor bone stock.

In proximal humeral fractures, a strategy for providing medial support using an endosteally placed segment of FA was introduced and demonstrated favorable outcomes.^[13–15]^ Inspired by this, a fixation strategy using FA with a locking plate was performed in patients with unfavorable conditions for bone stock. We performed 10 operations with FA in patients with poor bone stock or cortical bone defect at the nonunion site, and all patients exhibited good outcomes. However, several concerns related to allograft complications, such as disease transmission, infection, and immune reactions remain.^[22]^ Moreover, when considering subsequent surgeries, it is difficult to choose arthroplasty or intramedullary nail fixation due to the blocked intramedullary canal. Although we achieved successful results using this surgical method, the aforementioned issues should be carefully considered. More cases are required to validate the effectiveness of this surgical method. We will continue to observe and report these results consistently in the future.

In conclusion, the use of FA in conjunction with a locking plate for the fixation of nonunion peritrochanteric fractures with poor bone stock or cortical bone defects has yielded successful outcomes. Ongoing observations and reporting are essential for comprehensive validation and for addressing potential complications.

## Author contributions

**Conceptualization:** Won Chul Shin.

**Methodology:** Kyeong Baek Kim, Won Chul Shin.

**Visualization:** Min Uk Do, Hyun Tae Koo.

**Writing – original draft:** Min Uk Do.

**Writing – review & editing:** Min Uk Do, Won Chul Shin.
